# The PI3K regulatory subunit gene *PIK3R1* is under direct control of androgens and repressed in prostate cancer cells

**DOI:** 10.18632/oncoscience.243

**Published:** 2015-09-14

**Authors:** Jennifer Munkley, Karen E. Livermore, Urszula L. McClurg, Gabriela Kalna, Bridget Knight, Paul McCullagh, John McGrath, Malcolm Crundwell, Hing Y. Leung, Craig N. Robson, Lorna W. Harries, Prabhakar Rajan, David J. Elliott

**Affiliations:** ^1^ Institute of Genetic Medicine, Newcastle University, Newcastle-upon-Tyne, UK; ^2^ Northern Institute for Cancer Research, Newcastle University, Newcastle-upon-Tyne, UK; ^3^ Cancer Research UK Beatson Institute, Glasgow, UK; ^4^ Institute of Cancer Sciences, University of Glasgow, Glasgow, UK; ^5^ NIHR Exeter Clinical Research Facility, Royal Devon and Exeter NHS Foundation Trust, Exeter, UK; ^6^ Department of Pathology, Royal Devon and Exeter NHS Foundation Trust, Exeter, UK; ^7^ Exeter Surgical Health Services Research Unit, Royal Devon and Exeter NHS Foundation Trust, Exeter, UK; ^8^ Department of Urology, Royal Devon and Exeter NHS Foundation Trust, Exeter, UK; ^9^ Institute of Biomedical and Clinical Sciences, University of Exeter, Devon, UK

**Keywords:** PI3K signalling, PIK3R1, p85α, androgens, prostate cancer

## Abstract

Androgen receptor (AR) signalling and the PI3K pathway mediate survival signals in prostate cancer, and have been shown to regulate each other by reciprocal negative feedback, such that inhibition of one activates the other. Understanding the reciprocal regulation of these pathways is important for disease management as tumour cells can adapt and survive when either single pathway is inhibited pharmacologically. We recently carried out genome-wide exon-specific profiling of prostate cancer cells to identify novel androgen-regulated transcriptional events. Here we interrogated this dataset for novel androgen-regulated genes associated with the PI3K pathway. We find that the PI3K regulatory subunits *PIK3R1* (p85α) and *PIK3R3* (p55γ) are direct targets of the AR which are rapidly repressed by androgens in LNCaP cells. Further characterisation revealed that the PIK3CA p110α catalytic subunit is also indirectly regulated by androgens at the protein level. We show that *PIK3R1* mRNA is significantly under-expressed in prostate cancer (PCa) tissue, and provide data to suggest a context-dependent regulatory mechanism whereby repression of the p85α protein by the AR results in destabilisation of the PI3K p110α catalytic subunit and downstream PI3K pathway inhibition that functionally affects the properties of prostate cancer cells.

## INTRODUCTION

Prostate cancer (PCa), the most commonly-diagnosed malignancy in males [1], is characterised by its dependence on androgen receptor (AR) signalling. The initial treatment standard for patients with locally advanced or metastatic PCa is androgen deprivation therapy (ADT). This usually inactivates the AR, but after 2-3 years many patients develop castrate resistant PCa (CRPCa) where despite low serum testosterone levels AR signalling persists [2]. Progression to CRPCa may be the result of reactivation of AR signalling or reprogramming of the AR transcriptional landscape and has limited treatment options[3-5].

The phosphatidylinositol 3-kinase (PI3K) pathway is a key oncogenic signalling pathway in prostate cancer and has been shown to be altered in 42% of primary and up to 49% of metastatic cases[6, 7]. PIK3 signalling has a diverse array of functions including the regulation of cell survival, growth, proliferation, metabolism and angiogenesis [8]. The PI3K enzyme is an obligate heterodimer composed of a catalytic subunit (p110α) and one of a number of regulatory subunits [27]; the most common of which is derived from the p85α gene *(PIK3R1)*. Recently, reciprocal cross-talk between AR signalling and the PI3K pathway has been highlighted as a potential mechanism underlying CRPCa. AR signalling and the PI3K pathway have been shown to regulate each other by reciprocal negative feedback, such that inhibition of one pathway activates the other[9]. As both pathways mediate survival signals in prostate cancer tumour cells can adapt and survive when either single pathway is inhibited pharmacologically. Understanding the reciprocal regulation of these pathways is of critical importance in terms of disease management.

We recently carried out a global analysis of the PCa transcriptome to identify novel androgen-regulated transcriptional events[10-12]. In the light of evidence implicating cross-talk with the AR, we searched this dataset for novel androgen-regulated genes associated with PI3K signalling. Our data identifies a direct transcriptional link between the androgen receptor and PIK3 signalling pathways. We find that the *PIK3R1* gene is directly repressed by androgens and has decreased expression in clinical prostate cancer. Our data suggests a context-dependent regulatory mechanism whereby AR-mediated repression of the p85α protein results in destabilisation of the PI3K p110α catalytic subunit and downstream PI3K pathway inhibition.

## RESULTS

### The PI3K regulatory subunit genes *PIK3R1* and *PIK3R3* are direct targets of the AR

**Figure 1:**
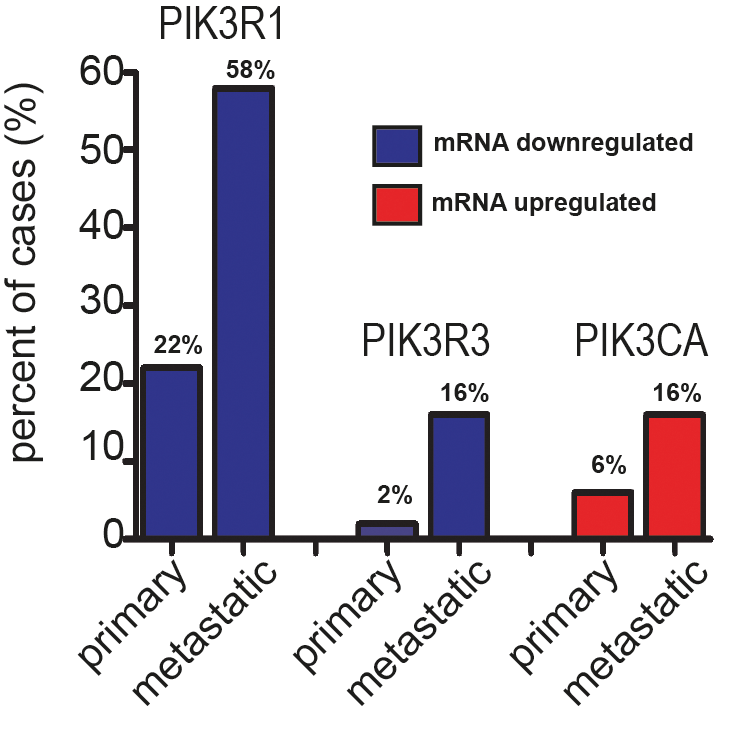
Expression of *PIK3R1, PIK3R3* and *PIK3CA* mRNA in 218 prostate cancer clinical samples as measured by Taylor et al. [6] *PIK3R1* mRNA is downregulated in 22% of primary and 58% of metastatic prostate cancer tissue samples (relative to normal prostate tissue).

Complete gene lists from our ExonArray dataset [10] were correlated with previously published mRNA expression data from 218 prostate tumours [6] with the aim of identifying novel androgen-regulated genes with roles in PI3K signalling. This highlighted two androgen-regulated subunits of PI3K encoded for by the *PIK3R1* and *PIK3R3* genes which were identified by Taylor et al. (2010) as being inactivated in 58% and 16% of metastatic prostate cancer tumours respectively [6] (Figure 1). Full gene lists were then uploaded to the web-based Ingenuity Pathway Analysis (IPA) software programme, and the IPA ‘Core Analysis’ function was used to identify additional androgen-regulated genes within pathways associated with *PIK3R1* and *PIK3R3* (Supplementary Figures 1 & 2). This identified an additional 34 genes which were validated as androgen-regulated in LNCaP cells by real-time PCR (Supplementary Table 1). The genomic loci of AR binding sites mapped by ChIP in LNCaP cells [13] were uploaded onto the UCSC genome browser. Two known AR binding sites within 100 kb of the *PIK3R1* gene and four AR binding sites within 50 kb of the *PIK3R3* gene were identified (Supplementary Table 2).

**Figure 2:**
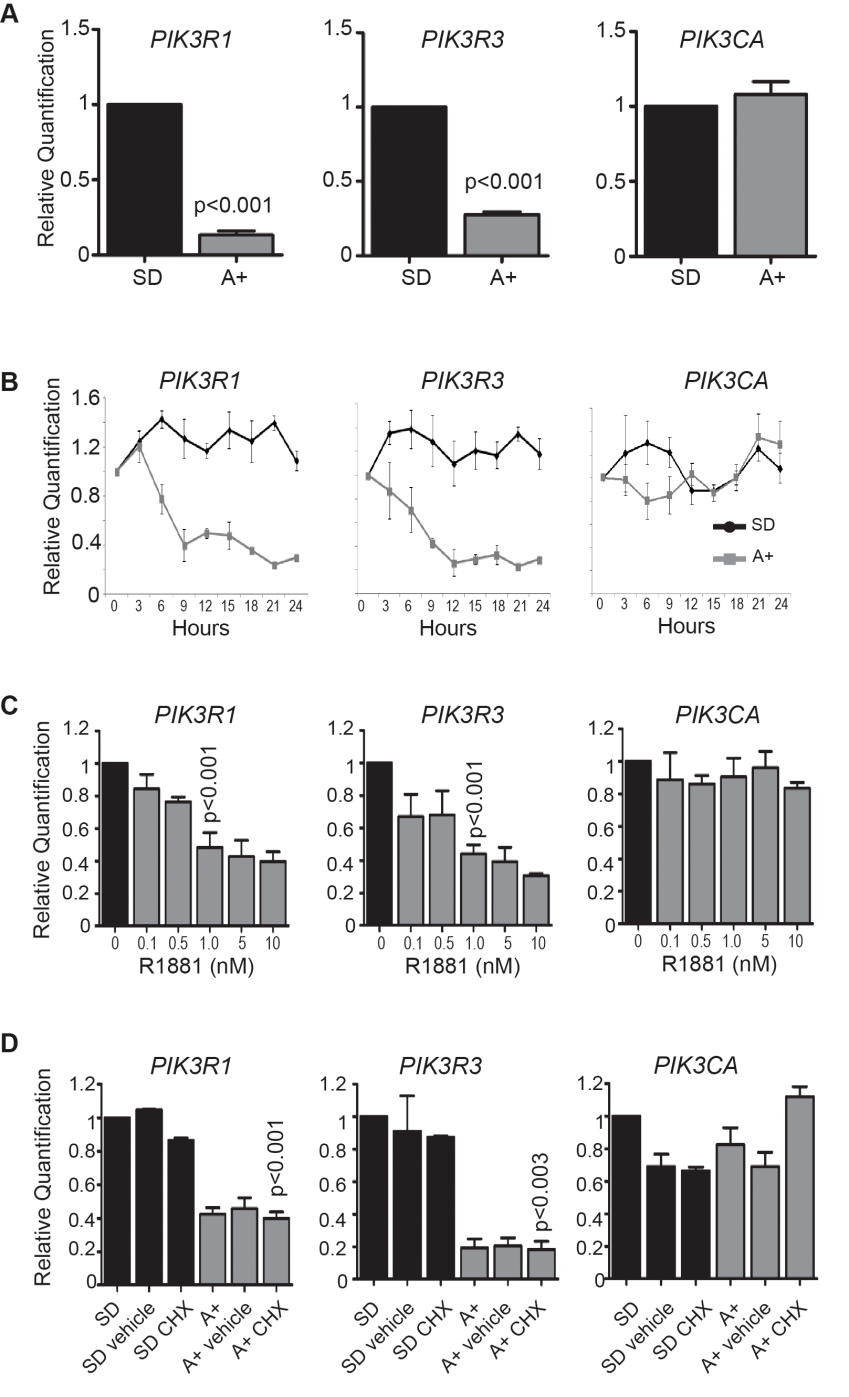
The PI3K regulatory subunit genes *PIK3R1* and *PIK3R3* are direct targets of the AR **(A)** LNCaP cells were cultured in medium supplemented with 10% dextran charcoal stripped FBS to produce a steroid deplete medium (SD). Following culture for 72 hours, cells were treated with 10 nM synthetic androgen analogue methyltrienolone (R1881) for 24 hours (A+). Relative expression of *PIK3R1*, *PIK3R3* and *PIK3CA* was detected by real-time PCR **(B)** Expression of *PIK3R1*, *PIK3R3* and *PIK3CA* mRNA in cells grown in steroid deplete (SD) or androgen (A+) treated conditions over a 24 hours period. The response to androgens was confirmed using PSA (KLK3) expression (not shown). **(C)** Repression of *PIK3R1* and *PIK3R3* is also evident in LNCaP cells treated with 0.1 to 100 nM of R1881. **(D)** The reduction in *PIK3R1* and *PIK3R3* mRNA expression in response to androgens is still seen in the presence of 1 μg/ml cycloheximide (CHX) as confirmed by real-time PCR.

To test whether the *PIK3R1* and *PIK3R3* genes might be under direct control of androgens through AR regulation, we examined *PIK3R1* and PIK3R3 expression in LNCaP cells grown in steroid deplete medium and cells treated with 10nM of the synthetic androgen analogue R1881 for 24 hours by real-time PCR. The *PIK3CA* gene which codes for the p110α catalytic subunit of PI3K was also studied. *PIK3R1* and *PIK3R3* mRNA expression were significantly reduced within 9 hours of treatment with 10nM R1881 (p<0.015), whereas there was no significant change in *PIK3CA* expression (Figure 2A,B). Repression of the *PIK3R1* and *PIK3R3* genes was also observed with a range of R1881 concentrations from 1nM to 100nM (p<0.01) consistent with this happening under physiological conditions within the prostate (Figure 2C). To test whether androgen-mediated suppression of *PIK3R1* and *PIK3R3* is a direct result of AR activity, we treated LNCaP cells with 10nM R1881 in the presence and absence of cycloheximide to inhibit *de novo* protein synthesis. Androgen mediated down-regulation of *PIK3R1* and *PIK3R3* was still observed in the presence of the protein synthesis inhibitor cycloheximide (p<0.04). Again, addition of cycloheximide had no effect on the expression of *PIK3CA* mRNA. The observation that *PIK3R1* and *PIK3R3* repression is not affected by inhibition of de novo protein synthesis indicates that this is likely to be directly mediated by the AR (Figure 2D). Although unlikely, it is still also possible that the regulation of *PIK3R1* and *PIK3R3* by androgens is mediated by an intermediate protein with a long half-life (which is the case for the AR).

### The p85α, p55γ and p110α proteins are repressed by androgens

**Figure 3:**
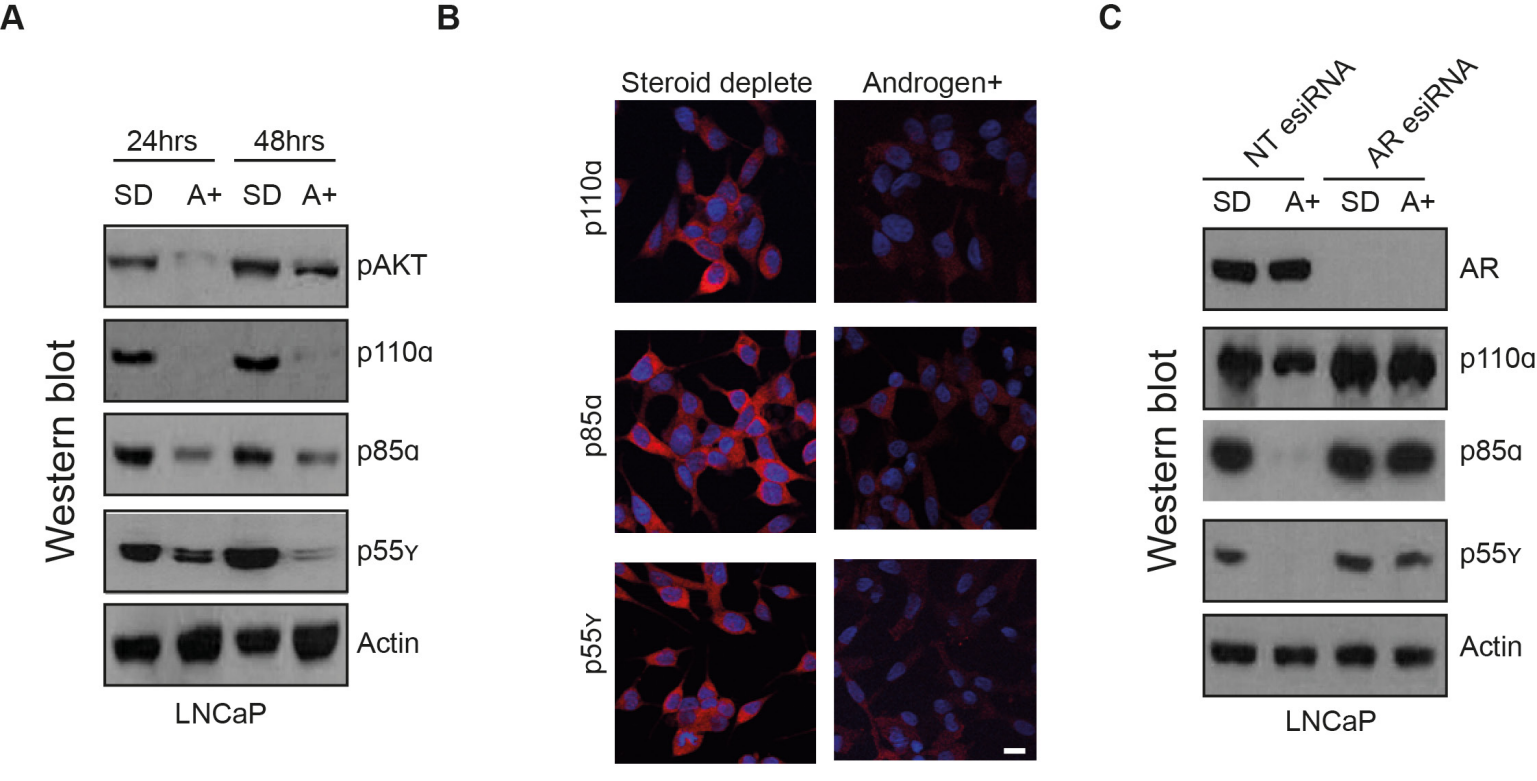
The p85α, p55γ and p110α proteins are repressed by androgens **(A)** Expression of p85α, p55γ and p110α protein is reduced in LNCaP cells treated with 10 nM R1881 for 24 and 48 hours as detected by western blot. Actin was used as a loading control. **(B)** Immunofluorescent staining of LNCaP cells grown in steroid depleted conditions indicates that p85α, p55γ and p110α are localised to the cytoplasm. Bar is 10 μM. **(C)** Depletion of AR protein in LNCaP cells by esiRNA shows that when the AR is depleted there is no reduction in p85α, p55γ or p110α protein in response to androgens. The specificity of the antibodies used was confirmed by detection of over-expressed protein and esiRNA mediated protein depletion (Supplementary Figure 3).

**Figure 4:**
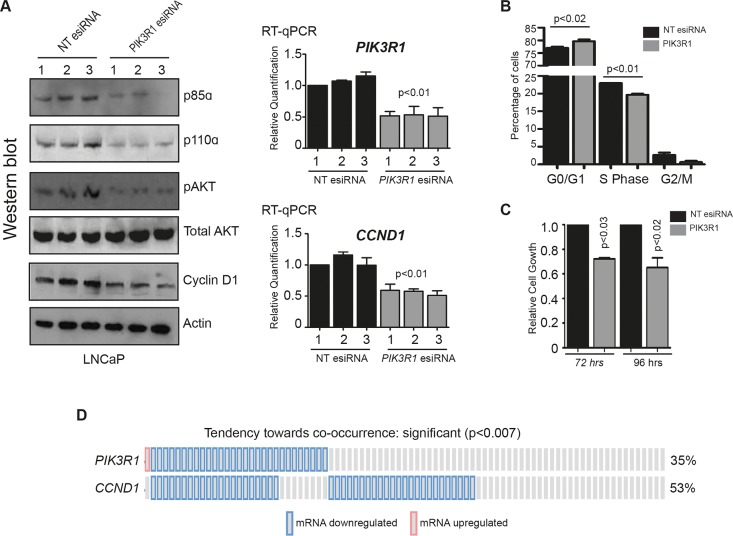
Depletion of p85α reduces pAKT in LNCaP cells and decreases cell proliferation **(A)** Depletion of *PIK3R1* / p85α in LNCaP cells by esiRNA was confirmed by western blotting (left panel) and by real-time PCR (right panel). Loss of p85α caused a reduction in the relative levels of p110α, pAKT (total AKT and actin were used as loading controls), and cyclin D. **(B)** Cell cycle analysis of LNCaP cells treated with *PIK3R1* esiRNA for 72 hours. **(C)** Relative cell number counts for LNCaP cells treated with *PIK3R1* esiRNA for 72 or 96 hours. **(D)** Analysis of data generated by Taylor et al. [6] for co-occurrence of *PIK3R1* and *CCND1* mRNA under-expression.

The above data shows that the p85α PI3K regulatory subunit gene *PIK3R1* and the p55γ subunit gene *PIK3R3* are direct transcriptional targets of the AR, whereas expression of the p110α PI3K catalytic subunit *PIK3CA* gene does not change with androgens. We next examined parallel expression of the p110α, p85α and p55γ proteins in LNCaP cells using western blotting and immunofluorescence. The expression of the p85α and p55γ proteins was repressed by treatment of 10nM R1881 for either 24 or 48 hours. Although we did not see any change in expression at the transcript level of the *PIK3CA* gene in response to androgens in LNCaP cells, at the protein level p110α was strongly repressed after treatment with androgens (Figure 3A,B). Confirming this effect on protein levels was mediated by the AR, we found that androgen-mediated repression of the p85α, p55γ and p110α proteins is prevented when cells are depleted of the AR using esiRNA (Figure 3C). The specificity of the antibodies used was confirmed by detection of over-expressed protein and detection of esiRNA mediated protein depletion (Figure 4A & Supplementary Figures 3A,B). Taken together, these results indicate that while the PI3K regulatory subunits p85α and p55γ are direct targets of the AR which are rapidly repressed by androgens, repression of the PI3K catalytic subunit p110α by androgens operates at the protein level only. Consistent with this pattern of repression of the PIK3 pathway, as observed previously [14] treatment of LNCaP cells with androgens also reduced expression levels of phosphorylated AKT (pAKT).

### The p110α PI3K catalytic subunit is stabilised by p85α

The above data predicted that AR-regulated suppression of p85α, p55γ and p110α in PCa cells may modulate PI3K signalling in response to androgens. To test this prediction, we depleted p85α and p55γ using esiRNA and examined the downstream effects on PI3K signalling in LNCaP cells. The p85α regulatory subunit has previously been shown to regulate and stabilise the PIK3CA p110α catalytic protein [15-17] 35]. Consistent with this, knockdown of p85α caused a reduction in p110α protein levels (Figure 4A). This result was confirmed by over-expression of p85α in HEK293 cells (which increased the level of p110α) (Supplementary Figure 3A). These findings support the repression of p110α by androgens being mediated by a mechanism involving reduction of its protein partner p85α (which is a direct target of the AR). In contrast, both depletion of p55γ by esiRNA in LNCaP cells, and over-expression of p55γ in HEK293 cells had no significant effect on p110α protein levels (Supplementary Figure 3A.B).

### Depletion of p85α reduces pAKT in LNCaP cells and decreases cell proliferation

We next examined phosphorylation of the serine kinase AKT (pAKT) as an indicator of endogenous PI3K activity [18]. Depletion of p85α in LNCaP cells reduced levels of pAKT (actin and total AKT levels were used as loading controls). In contrast, depletion of p55γ had no effect on pAKT. These results suggest that in LNCaP cells the depletion of p85α but not p55γ reduces PI3K activity (Figure 4A and Supplementary Figure 3B). Activated AKT kinase modulates the function of numerous substrates involved in cell cycle progression [19, 20], and we observed that depletion of p85α in LNCaP cells led to reduced levels of *cyclinD* mRNA and protein (Figure 4A). Consistent with these changes, cell cycle analysis of *PIK3R1* depleted cells indicated a significant reduction in the proportion of cells in S phase (p<0.01) and an increase in G_0_/G_1_ (P<0.02) (Figure 4B). We also observed significantly reduced cell growth after treatment with *PIK3R1* esiRNA (p>0.03) (Figure 4C). Taken together our results suggest that depletion of p85α in LNCaP cells results in downstream PI3K pathway inhibition that can functionally affect the properties of prostate cancer cells. As depletion of *PIK3R1* by esiRNA in LNCaP cells reduced expression of *CCND1* mRNA we tested whether patients with reduced *PIK3R1* also have reduced *cyclin D* expression. Consistent with this, re-analysis of the data generated by Taylor et al. [6] indicated a significant tendency towards co-occurance (p<0.007) for both down-regulation of *PIK3R1* and *cyclin D* (Figure 4D).

### *PIK3R1* is under-expressed in PCa

**Figure 5:**
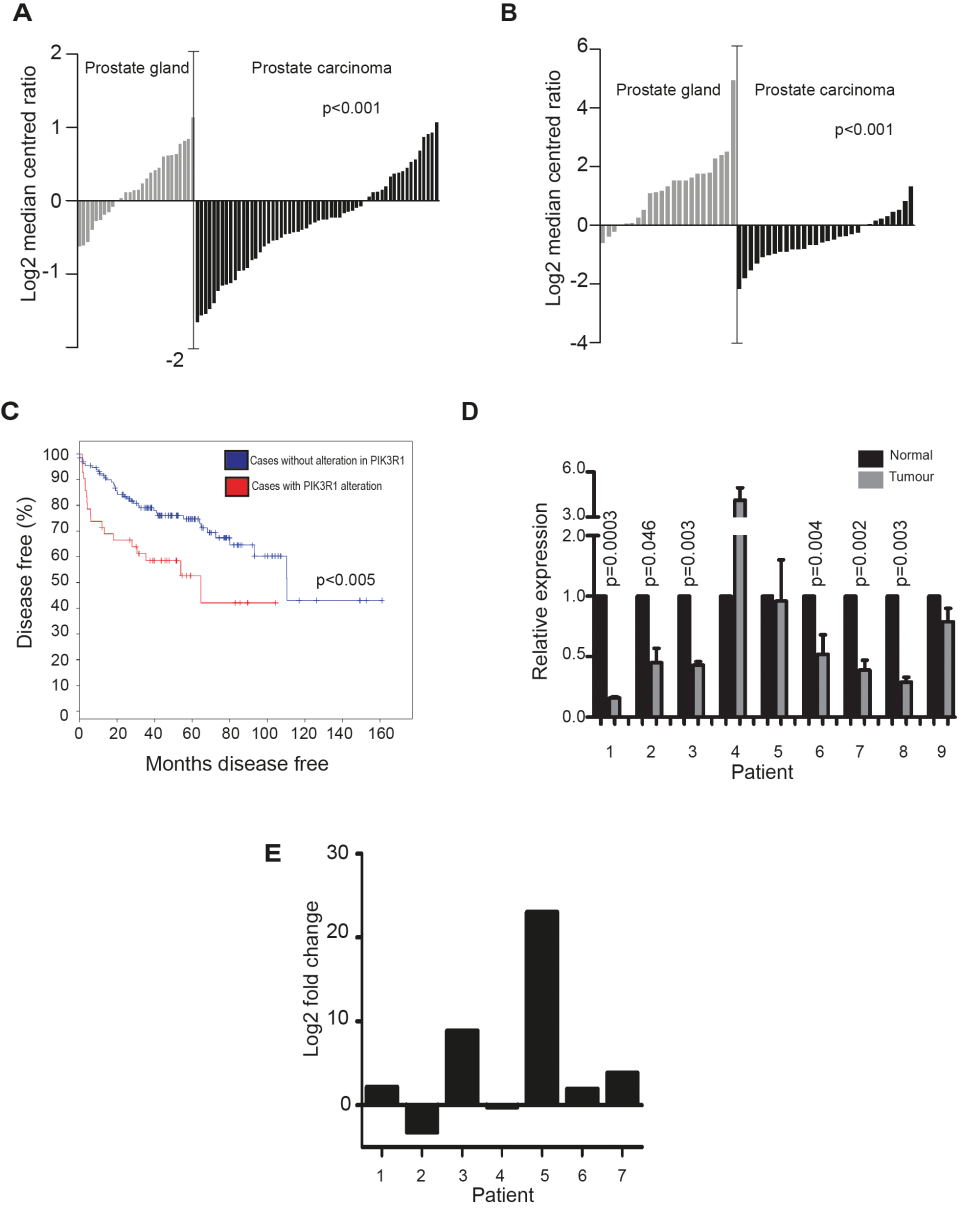
*PIK3R1* is under-expressed in PCa Expression of PIK3R1 in clinical PCa samples measured by **(A)** Grasso et al. [23] and **(B)** Tomlins et al. [21]. In both studies the expression of PIK3R1 mRNA is significantly reduced in prostate carcinoma relative to normal prostate gland tissue. Data was generated using Oncomine [30]. **(C)** Interrogation of previously published data [6] also showed a reduced rate of survival in patients which have an alteration in the *PIK3R1* gene. **(D)** Real-time PCR analysis of *PIK3R1* mRNA expression in matched normal and tumour tissue from 9 PCa patients. Expression of *PIK3R1* was significantly reduced in tumour relative to normal tissue in 6 out of the 9 patients studied (p<0.05). (E) Analysis of *PIK3R1* mRNA in previously published RNA-sequencing data [33] from 7 prostate cancer patients before and after androgen ablation therapy. PIK3R1 expression is increased in 5/7 patients following the removal of androgens (measured by log2 fold change of RNA-Seq reads detected).

We carried out a further meta-analysis of 747 prostate cancer tumours using data from 10 previously published studies[6, 21-29]. Analysis of *PIK3R1*, *PIK3R3* and *PIK3CA* mRNA expression in these datasets revealed that *PIK3R1* was significantly down-regulated in PCa tumours relative to normal tissue in 9 of the 10 studies. *PIK3R3* and *PIK3CA* were significantly altered in 3/10 studies and 1/10 respectively (Supplementary Table 3). Analysis of the 122 samples studied by Grasso *et al*. [23] identified *PIK3R1* as −1.428 fold under-expressed in prostate carcinoma relative to normal prostate tissue (p=4.99E-5) and placed *PIK3R1* in the top 10% of under-expressed genes (Figure 5A). Similarly, analysis of 101 samples studied by Tomlins *et al.* [21] found that expression of *PIK3R1* was reduced by −3.266 fold (p=5.87E-7) in prostate carcinoma relative to normal tissue, and in this dataset *PIK3R1* was in the top 1% of under-expressed genes (Figure 5B) (data was generated using Oncomine [30]). Interrogation of previously published data [6] also showed reduced survival in patients where the *PIK3R1* gene is altered (median months disease free is reduced from 110 to 65 (p<0.005) (Figure 5C). We also analysed expression of *PIK3R1* mRNA in 9 prostate tumour tissue samples relative to matched normal tissue from the same patient. *PIK3R1* expression was significantly reduced in 6/9 prostate tumour tissue samples when compared with matched normal tissue from the same patient (Figure 5D). Analysis of *PIK3R3* and *PIK3CA* expression in the same sample sets indicated no significant differences (Supplementary Figure 4). The above data indicates that PIK3R1 expression is repressed by the androgen receptor in LNCaP cells in response to androgens. In order to see if reciprocal changes might occur in patients before and after androgen deprivation therapy we analysed previously published RNA-Sequencing data from 7 PCa patients [13]. In 5/7 patients expression of *PIK3R1* was increased after androgens were depleted (Figure 5E) consistent with the change in PIK3R1 expression in response to androgens being increased in patients in response to anti-androgen treatment. Taken together, these results indicate that PIK3R1 is under-expressed in PCa.

## DISCUSSION

**Figure 6:**
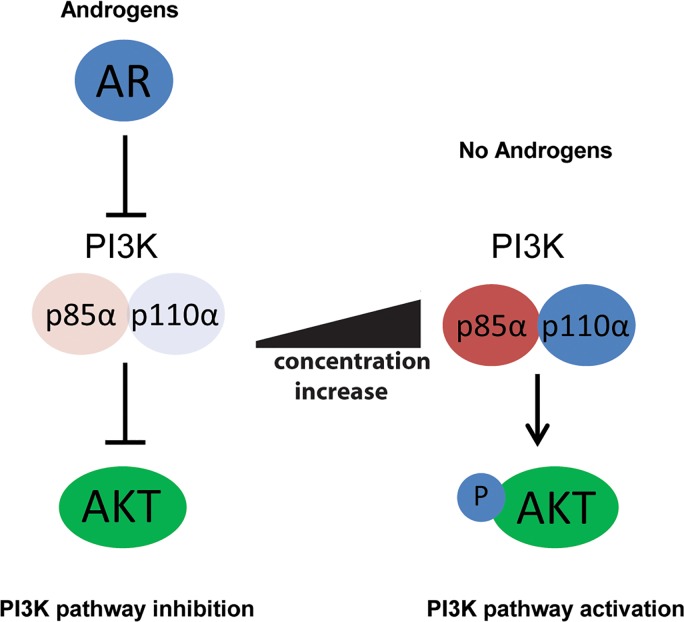
Model: AR-mediated repression of the p85α protein results in destabilisation of the PI3K p110α catalytic subunit and downstream PI3K pathway inhibition Androgen receptor (AR) signalling and the PI3K pathway have been shown to regulate each other by reciprocal negative feedback in PTEN negative cells. We show that the PI3K regulatory subunit *PIK3R1* (p85α) is a direct target of the AR which is rapidly repressed by androgens in prostate cancer cells. The data presented suggests a context-dependent regulatory mechanism whereby repression of the p85α protein by the AR results in destabilisation of the PI3K p110α catalytic subunit downstream PI3K pathway inhibition.

Here we show that expression of the PI3K regulatory subunit p85α protein-encoding gene *PIK3R1* is under direct control of androgens and is reduced in prostate carcinoma tissue relative to the normal prostate gland. Our data identifies a direct transcriptional link between the androgen receptor and PI3K signalling pathways and suggests a context-dependent regulatory mechanism whereby repression of the p85α protein by the AR results in destabilisation of the PI3K p110α catalytic subunit and downstream PI3K pathway inhibition (Figure 6). A reciprocal regulation between PI3K and AR and signalling pathways has been previously implicated in prostate tumorigenesis [7][14, 33]. As tumour cells can adapt and survive when either single pathway is inhibited pharmacologically, understanding the reciprocal regulation of these pathways further is crucial for disease management [31]. Carver et al. [7] showed that PI3K signalling inhibits AR signalling via feedback inhibition of human epidermal growth factor 2/3 (HER2/3), and that AR signalling down-regulates PI3K signalling through FK506-binding protein-5 (FKBP-5) mediated stabilisation of the AKT phosphatase PHLPP [7]. The new data reported here thus indicate that these two pathways are even more closely linked at the level of transcriptional control.

*PIK3R1* under-expression has previously been shown to be an independent prognostic marker in breast cancer [29] and low *PIK3R1* expression has been linked to high grade lung cancer [30]. Liver specific loss of *PIK3R1* in a murine model results in the development of aggressive hepatocellular cancer [13], and loss of *PIK3R1* mRNA in the MCF-7 breast cancer cell line is associated with a more invasive phenotype [32]. Studies have also suggested that *PIK3R1* expression negatively influences glioblastoma tumour growth and patient survival [33]. The role of p85α in the PI3K pathway is complex. In contrast to the studies discussed above, *PIK3R1* has also been implicated as an oncogene in endometrial, ovarian and colon tumours [28] [16]. Previous studies have shown that optimal signalling through the PIK3 pathway depends on a critical molecular balance between the regulatory and catalytic subunits; haploinsufficiency of PIK3R1 can result in PI3K pathway activation, whereas homozygous depletion inhibits the pathway [22][31, 34]. Point mutations in and deletions of the *PIK3R1* gene have been reported in up to 20% of endometrial cancer cases and in 2.2% of breast cancers [16, 36] [29]. Analysis of organoid cultures derived from patients with advanced prostate cancer detected mutations in *PIK3R1* in 2/7 patients [30]. Taylor *et al.* [6] did not identify any *PIK3R1* mutations in their study of 218 PCa tumours. However, a recent study by Robinson *et al*. [7] identified *PIK3R1* mutations and deletions in 4.7% and 1.3% of metastatic CRPCa respectively. Together with our data this shows that down-regulation of *PIK3R1* mRNA/p85α is more common in prostate tumours than mutation of the gene, and the effect of p85α on the PI3K pathway and tumour development is likely to be tissue and context-dependent and determined by the relative activities of p110α, p85α and PTEN.

Our work demonstrates a direct transcriptional link between AR signalling and the PI3K pathway mediated by AR-dependent repression of *PIK3R1.* Recent work has demonstrated a dynamic interplay between PI3K and AR signalling in resistance to ADT [31, 32]. Activation of PI3K signalling as a result of treatments targeting the AR may therefore enable prostate cancer cells to survive and proliferate without androgens. This concept is underlined by the finding that androgen inhibition actually accelerates progression to invasive PCa in PTEN-deficient mice [34]. These findings and the work described in this study support a route involving combinatorial inhibition of AR and PI3K signalling to significantly reduce progression to CRPCa [35].

## METHODS

### IPA pathway analysis

Gene lists from Rajan et al. [10] were uploaded to the web-based Ingenuity Pathway Analysis (IPA; Ingenuity Systems) software programme, and the “Core Analysis” function was used to study direct and indirect regulatory relationships between genes and their known biological functions.

### Antibodies

The following antibodies were used in the study: p85α mouse antibody (Abcam, ab22653), anti p55γ rabbit antibody (Sigma, HPA005751), p110α rabbit antibody (Cell Signalling, 4249), Total AKT rabbit antibody (Santa Cruz, sc-8312), anti-phospho-AKT1 (pSer473) rabbit antibody (Sigma, SAB4300042), anti-Cyclin D1 (Abcam ab21699), anti-AR mouse antibody (BD Bioscience, 554226), anti-FLAG mouse monoclonal (F3165, Sigma), normal rabbit IgG (711-035-152 Jackson labs) and normal mouse IgG (715-036-150 Jackson labs).

### RT-qPCR

Cells were harvested and total RNA extracted using TRI-reagent (Invitrogen, 15596-026), according to the manufacturer's instructions. RNA was treated with DNase 1 (Ambion) and cDNA was generated by reverse transcription of 500ng of total RNA using the Superscript VILO cDNA synthesis kit (Invitrogen, 11754-050). Quantitative PCR (qPCR) was performed in triplicate on cDNA using SYBR® Green PCR Master Mix (Invitrogen, 4309155) using the QuantStudio™ 7 Flex Real-Time PCR System (Life Technologies). Samples were normalised using the average of three reference genes: GAPDH, β –tubulin and actin. All primer sequences are listed in Supplementary Table 4.

### DNA constructs

For creation of the Flp-In™-293 stable cell line *PIK3R1* and *PIK3CA* and were cloned into pCDNA5 using *Bam*H1 and *Not*1. *PIK3R3* was cloned into pCDNA5 using *Not*1 and *Xho*1.

### Cell culture

Cell culture and androgen treatment of cells was as described previously [10-12]. All cells were grown at 37°C in 5% CO2. LNCaP cells (CRL-1740, ATCC) were maintained in RPMI-1640 with L-Glutamine (PAA Laboratories, R15-802) supplemented with 10% Fetal Bovine Serum (FBS) (PAA Laboratories, A15-101). For androgen treatment of LNCaP cells, medium was supplemented with 10% dextran charcoal stripped FBS (PAA Laboratories, A15-119) to produce a steroid-deplete medium. Following culture for 72 hours, 10nM synthetic androgen analogue methyltrienolone (R1881) (Perkin–Elmer, NLP005005MG) was added (Androgen +) or absent (Steroid deplete) for the times indicated. Where indicated, LNCaP cells were pre-treated for 1 hour with vehicle (dimethylsulfoxide; DMSO) (Sigma, C1988) or 1 μg/ml cycloheximide (Sigma, D2438) prior to addition of 10 nM R1881 for 24 hours as previously described [16]. Flp-In™-293 cells (R750-07, Invitrogen) were maintained in DMEM GlutaMax (Invitrogen, 10566-040), supplemented with 10% FBS (PAA Laboratories, A15-101) and stable cell lines generated using the Flp-In T-Rex Core Kit (K6500-01, Invitrogen) according to the manufacturer's instructions. Protein expression was induced using 1 μg/ml tetracycline (T7660, Sigma) for 72 hours.

### esiRNA

esiRNAs *PIK3R1* and *PIK3R3* were obtained from Sigma-Aldrich (EHU151811 and EHU123491).

### Cell cycle analysis

Cell cycle analysis was carried out using the TaliR Cell Cycle Kit (Life Technologies A10798) and the TaliR Image-based Cytometer. The data was then analysed using ModFit. Relative cell numbers following esiRNA treatment were determined using the TaliR Image-based Cytometer (Life Technologies).

### Clinical samples

Our study made use of RNA from 32 benign samples from patients with benign prostatic hyperplasia (BPH) and 17 malignant samples from transurethral resection of the prostate (TURP) samples. Malignant status and Gleason score were obtained for these patients by histological analysis. We also analysed normal and matched PCa tissue from 9 patients obtained by radical prostectomy. The samples were obtained with ethical approval through the Exeter NIHR Clinical Research Facility tissue bank (Ref: STB20). Written informed consent for the use of surgically obtained tissue was provided by all patients.

## SUPPLEMENTARY DATA TABLES AND FIGURE


