# Supplementary material for: The PI3K regulatory subunit gene PIK3R1 is under direct control of androgens and repressed in prostate cancer cells
Source: Oncoscience. 2015 Sep 14;2(9):755–64. doi: 10.18632/oncoscience.243 (PMC4606009; doi:10.18632/oncoscience.243)

Supplementary Data

Supplementary Table 1

Additional *PIK3R1* and *PIK3R3* genes identified by IPA pathway analysis validated as androgen regulated in LNCaP cells by real-time PCR

Supplementary Table 2

Previously published known AR binding sites within 100 kb of the *PIK3R1* gene and four AR binding sites within 50 kb of the *PIK3R3* gene.

Supplementary Table 3

Meta-analysis of *PIK3R1*, *PIK3R3*, *PIK3CA* and *CCND1* mRNA expression in 728 prostate cancer tumours using data from 10 previously published studies [6,21-28]. Data was generated using Oncomine [30].

Supplementary Table 4

Primer sequences used

Supplementary Figure 1: IPA Pathway Analysis of genes associated with *PIK3R1*.

Gene expression changes in LNCaP cells cultured in the presence or absence of androgens for 24 hours. Gene lists from Rajan et al. [10] were uploaded to the web-based Ingenuity Pathway Analysis (IPA; Ingenuity Systems) software programme, and used to identify novel androgen-regulated genes associated with *PIK3R1*. Genes up-regulated by androgens are highlighted in red, and down-regulated genes are in green. Expression changes for 34 of these genes were validated by real-time PCR (Supplementary Table 1).

Supplementary Figure 2: IPA Pathway Analysis of genes associated with *PIK3R3*.

Gene expression changes in LNCaP cells cultured in the presence or absence of androgens for 24 hours. Gene lists from Rajan et al. [10] were uploaded to the web-based Ingenuity Pathway Analysis (IPA; Ingenuity Systems) software programme, and used to identify novel androgen-regulated genes associated with *PIK3R3*. Genes up-regulated by androgens are highlighted in red, and down-regulated genes are in green.

Supplementary Figure 3: *Over-expression of p85α in HEK293 cells stabilises p110α.*

(A) Over-expression of p85α, p55γ and p110α in Flp-In HEK293 cells was induced by addition of 1 μg/ml tetracycline to the tissue culture medium for 72 hours and detected by western blotting using its corresponding antibody. This confirmed the specificity of our p85α, p55γ and p110α antibodies and showed that an increase in p85α protein stabilises p110α **(B)** Depletion of PIK3R3 / p55γ in LNCaP cells by esiRNA was confirmed by western blotting (upper panel) and by real-time PCR (lower panel).

Supplementary Figure 4: PIK3R3 and PIK3CA expression in clinical samples.

(A) Real-time PCR analysis of *PIK3R1* mRNA expression in 32 BPH and 17 PCa tissue samples, and from 9 matched normal and tumour tissue pairs (normalised to 3 housekeeping genes). **(B)** Real-time PCR analysis of *PIK3R1* mRNA expression in 32 BPH and 17 PCa tissue samples, and from 9 matched normal and tumour pairs(normalised to 3 housekeeping genes).

Supplementary Table 1

PI3K pathway **RT_PCR**

Up-regulated

	Fold change*	SE	P value	Taylor *et al.* Fold change	Taylor *et al.* P value
LDLR	6.19	0.87	3.98E-03		not sig.
RAB3B	5.02	0.45	8.40E-04	1.659	1.45E-04
HMGCR	3.28	0.73	0.03521		not sig.
VEGFA	4.23	0.34	6.40E-04		not sig.
NR4A1	13.33	3.95	0.03547	1.220	0.027
SOCS2	9.75	1.56	4.96E-03	1.147	8.00E-03
MTOR	4.25	0.51	3.07E-03	1.215	2.14E-04
ERRFI1	3.49	0.86	0.04492	1.290	2.00E-03
VLDLR	2.20	0.28	0.01313	1.258	3.00E-03
SCR	1.05	0.15	0.74037		not sig.
ANGPT2	31.82	2.80	3.90E-04		not sig.
CALU	7.55	0.85	1.54E-03		not sig.
ORM1	446.31	57.45	1.49E-03		not sig.
LIFR	13.95	3.40	0.01896	1.303	9.00E-03
GHR	2.65	1.01	0.17985	1.095	2.00E-03
MERTK	14.00	3.01	0.0125		not sig.
SEC14L2	8.46	0.74	5.50E-04		not sig.
CSK	1.96	0.42	0.08213	1.083	6.00E-03
ARFIP2	2.08	0.31	0.02542	1.216	9.94E-05
TBC1D4	4.64	0.41	9.30E-04	1.467	8.65E-06

Down-regulated

	Fold change*	SE		Taylor et al. Fold change	Taylor et al. P value
TLL1	0.38	0.12	5.73E-03	-1.145	2.25E-06
F2R	0.31	0.09	1.80E-03		not sig.
SI	0.02	0.01	7.23E-09		not sig.
ADD3	0.21	0.09	8.30E-04	-1.284	0.002
SDC4	0.29	0.04	4.32E-05	-1.471	2.13E-09
KLF10	0.06	0.00	1.50E-08		not sig.
DEPDC6	0.16	0.02	1.14E-06	-1.475	9.00E-06
SORBS2	0.16	0.02	3.67E-06		not sig.
PIK3C2B	0.58	0.02	4.19E-05		not sig.
ASAP2	0.56	0.23	0.1304		not sig.
SDC2	0.31	0.07	4.70E-04	-1.272	1.33E-07
HSPG2	0.49	0.11	9.21E-03	-1.370	1.07E-06
ST5	0.38	0.11	4.90E-03	-1.704	1.93E-13
AMPK (PPL	0.67	0.16	0.1088	-1.081	2.36E-04

* Fold change = relative quantification of androgen treated cells normalised to steroid deplete (set as 1)

Supplementary Table 2

**PIK3R1 gene**		start	end	
	chr5:	67,511,584	67,597,649	
**PIK3R1 ChIP-seq peaks**		start	end	distance
		67419340	67419849	**92,244**
		67424772	67425507	**86,812**

**PIK3R3 gene**		start	end	
	chr1:	46505812	46598708	
**PIK3R3 ChIP-seq peaks**		start	end	distance
		46634665	46635340	**35957**
		46634214	46634617	**35909**
		46634665	46635340	**36632**
		46640496	46640802	**42094**

**PIK3CA gene**		start	end	
	chr3:	178,866,311	178,952,497	
**PIK3CA ChIP-seq peaks**		start	end	distance
		178,791,302	178791302	**75,009**

Supplementary Table 3

Cancer v normal analysis fold changes
747 samples

	Tomlins et al. 2007	Lapointe et al. 2004	Grasso et al 2012
	101 samples	112 samples	122 samples
	Prostate carcinoma epithelia v normal	Prostate carcinoma v normal	Prostate carcinoma vs normal
**PIK3R1**	-3.266 p=5.87E-7	-1.561 p=1.89E-7	-1.428 p=4.99E-5.
**PIK3R3**	1.771 p=0.999	1.109 p=0.966	1.275 p=0.934
**PIK3CA**	1.581 p=0.063	1.058 p=0.878	-1.079. p=0.943.
**CCND1**	1.039 p=0.569.	-1.061 p=0.212.	-1.425 p=1.92E-7.

	Luo et al. 2002	Arredouani et al. 2009	Vanaja et al. 2003
	30 samples	21 samples	40 samples
	Prostate carcinoma vs normal	Prostate carcinoma vs normal	Prostate adenocarcinoma vs normal
**PIK3R1**	-6.355 p=0.034.	p=0.002. -1.972.	p=0.253. -1.118
**PIK3R3**	1.156 p=0.406.	p=0.709. 1.070	-1.174. p=0.019.
**PIK3CA**	2.451 p=0.106	p=0.708.-1.049	-1.087. p=0.043
**CCND1**	-1.296 p=0.091.	-1.338 p=0.002.	-1.052 p=0.668.

	Taylor et al 2010	Varmabally et al 2007
	185 samples	19 samples
	Prostate carcinoma vs normal	Prostate carcinoma vs normal
**PIK3R1**	-1.408 p=1.73E-6	-1.446 p=0.002
**PIK3R3**	1.183 p=0.999	p=0.474.-1.040
**PIK3CA**	-1.072 p=0.879.	p=0.937.-1.181
**CCND1**	-1.375 p=3.08E-11.	-1.865 p=1.33E-4.

	Magee et al. 2001	Singh et al. 2002
	15 samples	102 samples
	Prostate carcinoma vs normal	Prostate carcinoma vs normal
**PIK3R1**	p=0.006. -1.657.	p=0.021. -1.537.
**PIK3R3**	p=0.287. -2.676.	-2.924. p=5.91E-5
**PIK3CA**	-1.895. p=0.883.	p=0.508. -1.003
**CCND1**	-1.100 p=0.227.	-1.416 p=0.015.

Supplementary Table 4

Primer sequences used

Gene	Forward	Reverse
PIK3R1	GATTCTCAGCAGCCAGCTCTGAT	GCAGGCTGTCGTTCATTCCAT
PIK3R3	CGTGTGCCATTTGTTTTGAC	ATTCCAGAGCCAAGCATCAT
PIK3CA	GAGAGGGGAATGAAAAGGAGA	ATCATGAATCTCACCCAGACG
GAPDH	AACAGCGACACCCATCCTC	CATACCAGGAAATGAGCTTGACAA
Actin	CATCGAGCACGGCATCGTCA	TAGCACAGCCTGGATAGCAAC
B-tubulin	CTTCGGCCAGATCTTCAGAC	AGAGAGTGGGTCAGCTGGAA
CCND1	CCGCTGGCCATGAACTACCT	ACGAAGGTCTGCGCGTGTT
LDLR	AAGGACACAGCACACAACCA	CAAAGGAAGACGAGGAGCAC
RAB3B	ACGAGAAGCGGGTGAAACT	CCAGTCTTGGACAGCATTGA
HMGCR	GACCTTTCCAGAGCAAGCAC	ACCAACTCCAATCACAAGACA
VEGFA	AAGGAGGAGGGCAGAATCAT	CATCAGGGGCACACAGGAT
NR4A1	GCTACCTTCAAAACCCAAGC	CAGCATCTTCCTTCCCAAAG
SOCS2	TGCCTTGCCTTCTTAGGTTC	GCTTGGTTCCTTCCCACTTC
MTOR	ACGCTGTCATCCCTTTATCG	GCTCAAACACCTCCACCTTC
ERRFI1	GGAGCAGTCGCAGTGAGTTT	AATAAGAAGAGGGGGCAAGC
VLDLR	GCACAAATGGTCGCTGTATT	GCACTGTTCTGGGCTTTCAT
SCR	TCCTGGTGGGAGAGAACCTG	CCGACTTGATGGTGAAGC
ANGPT2	GGGAAGGGAATGAGGCTTAC	GGTTGGCTGATGCTGCTTAT
CALU	TGAGCAAACCCACAGAAAAG	AGCACCCAAGAAGGCATCAT
ORM1	CCAACAAGACAGAGGACACG	TGGACATTCAGGTAGGTGGTG
LIFR	CCACCTTCCAAAATAGCGAGT	GTTCCGACCGAGACGAGTTA
GHR	AAGTGAGATGGGAAGCACCA	CCAGAGTTTCGTTGTTTGGA
MERTK	CTGAAAGTGGCAGTGAAGACC	GACATTTGGGTGGCTGAAGT
SEC14L2	ACCTCATCAAACCCTTCCTG	GTGCCCCCATACTCCACA
CSK	GCCCAGGATGAGTTCTACCG	ACTTGACGGCGACTTTGTTC
ARFIP2	CCATCGGGACAAGTATGAGAA	AAAGTAGGCGGACACAGCAT
TBC1D4	GCAATGAGTCCCTAAGTGTGG	CGTTCCTGTCCAATCCATCT
TLL1	GGCAATCTCTATCGGCAAGA	CCTGGCTGGATGTTTTCTCT
F2R	GCCAGAATCAAAAGCAACAA	CTCCTCATCCTCCCAAAATG
SI	TTCCTTGGTGCTTCTTCGTT	CGATTGGGTGTCTGATTTTG
ADD3	ACCAGTCCCCGAACCAAA	GGTCCTGCTGTTTTCAAGTCA
SDC4	CCACCGAACCCAAGAAACTA	ATGCCACCCACAATCAGAG
KLF10	CAATGAAAGCAGCCAGCAT	GAGGAAGGCACAGAAAAGTCA
DEPDC6	GTCCGTGCTGAAGAGACCTG	CCGTTGACAGAGACGACAAA
SORBS2	CAAATAAGCCACAGCGTCCT	CCACAAACCAGCCGTCAT
PIK3C2B	AATGCTGTCCCCCTCAAACT	GTCCTGGCGAAGGTCGTC
ASAP2	ATCCGAAAAGTGTGGCAGAA	TGGCAGGTTAGCAGGTTGAG
SDC2	CCAGCCGAAGAGGATACAAA	ATGCGATACACCAACAGCAG
HSPG2	GTGCTCATCCAGACCGTGTA	GACAAGCCAGAATAGCCAATG
ST5	TACCCCTCTTCTCCCACTGA	TCCTGCTCTTCGGCTCTTTA
AMPK (PPLAB.	AGGCACCAGAGAAGGAGGAA	CAGTTGTTGAAGGACCCAGA

Supplementary Figure 1

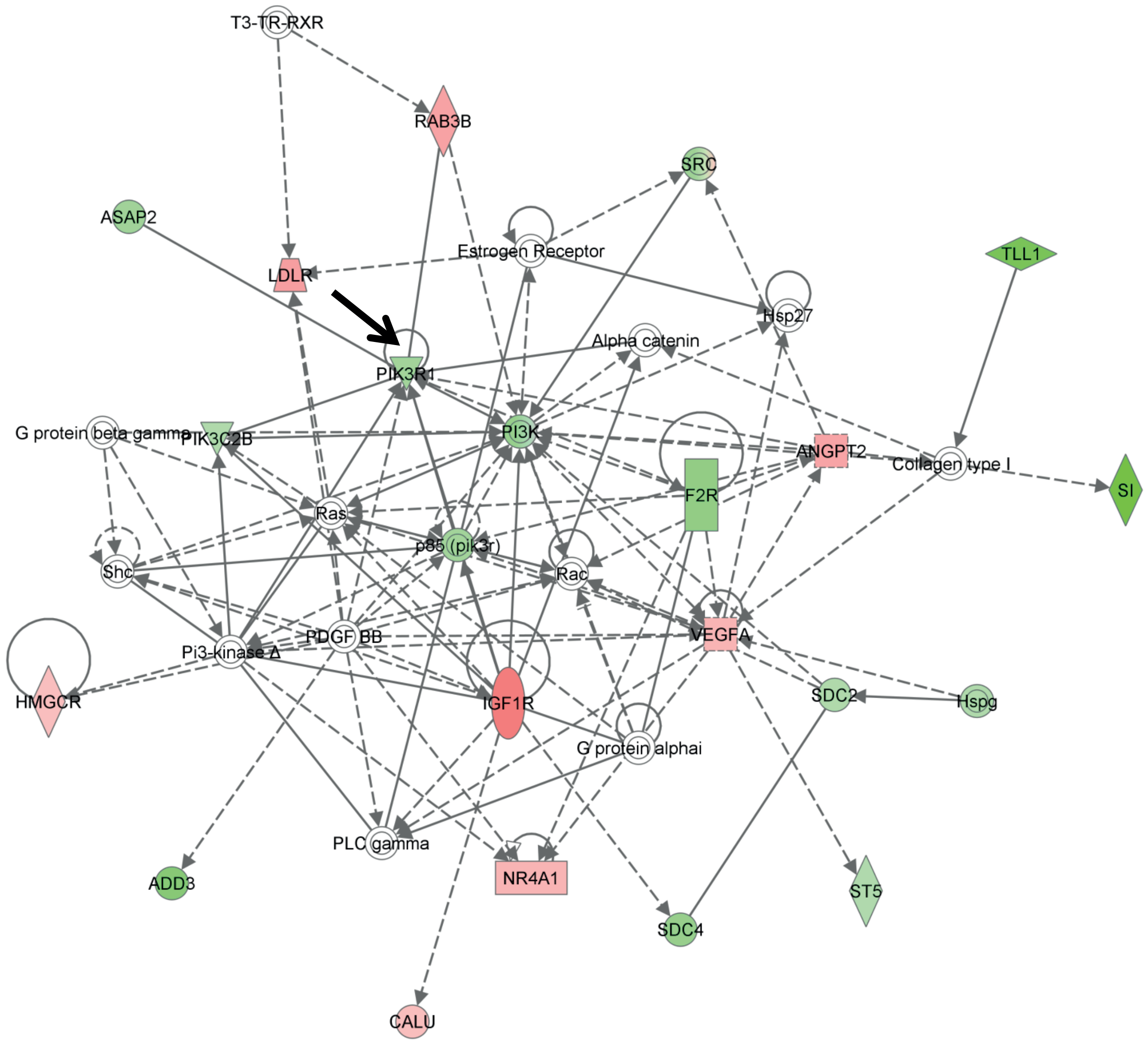

Supplementary Figure 2

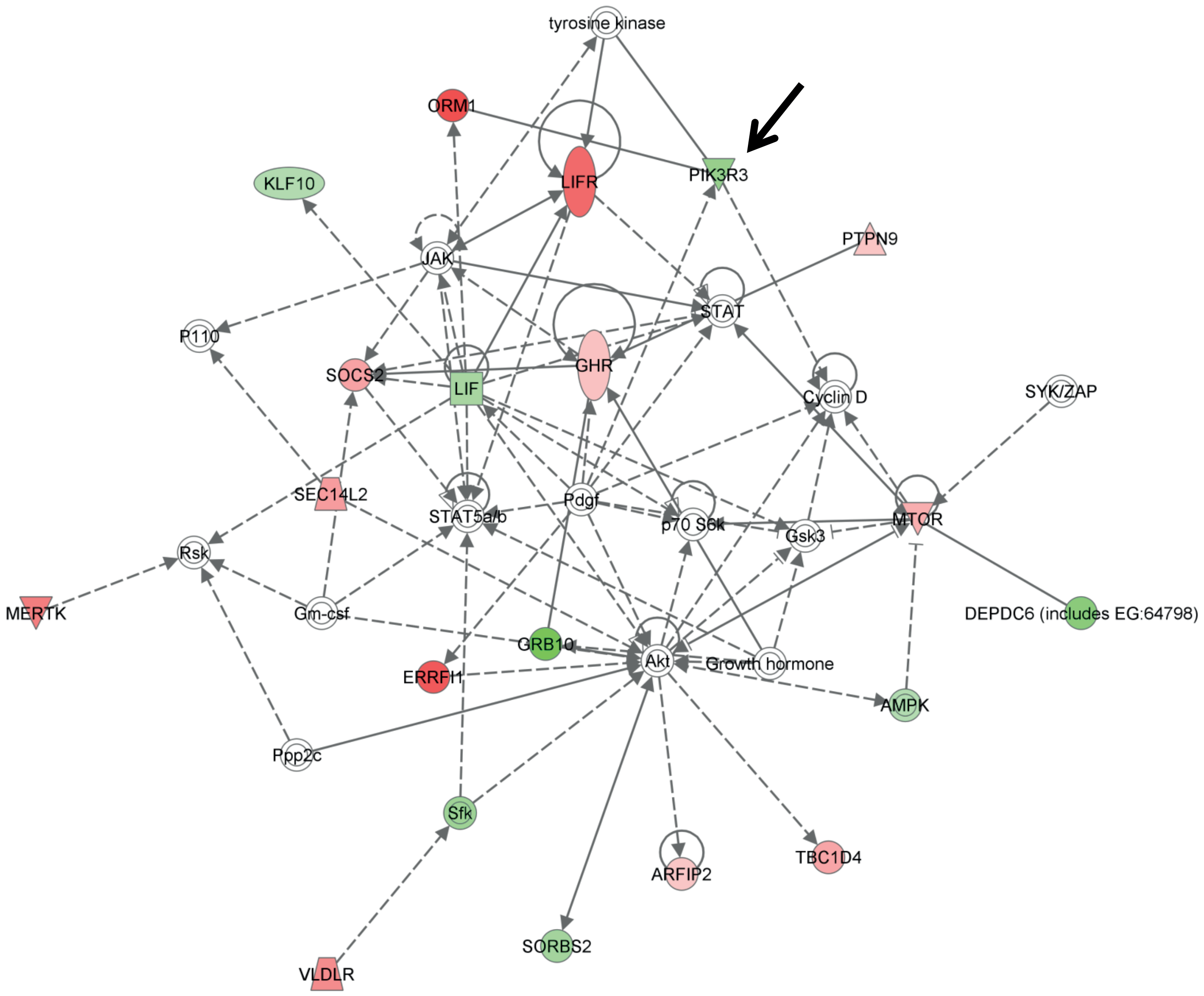

Supplementary Figure 3

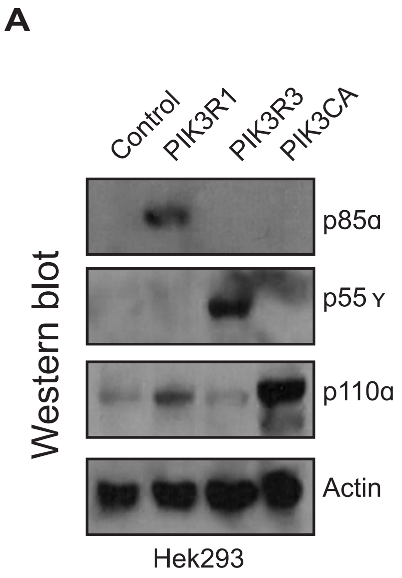

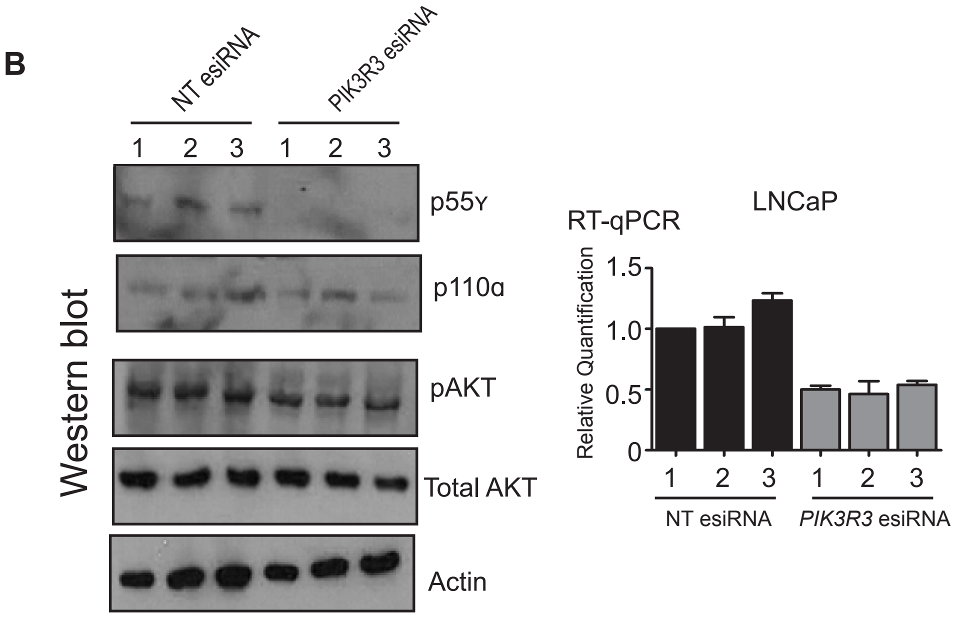

Supplementary Figure 4

A

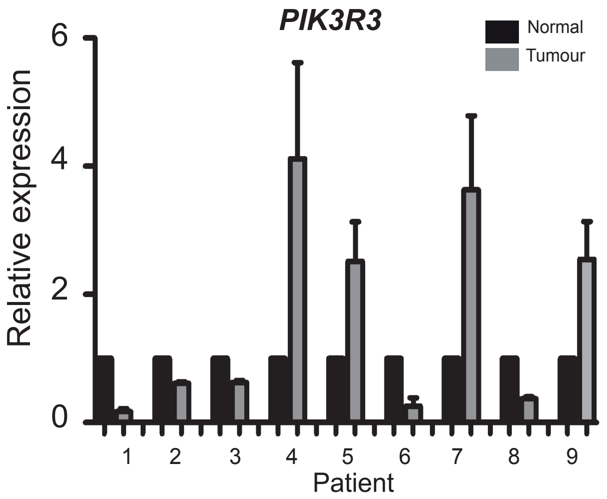

B

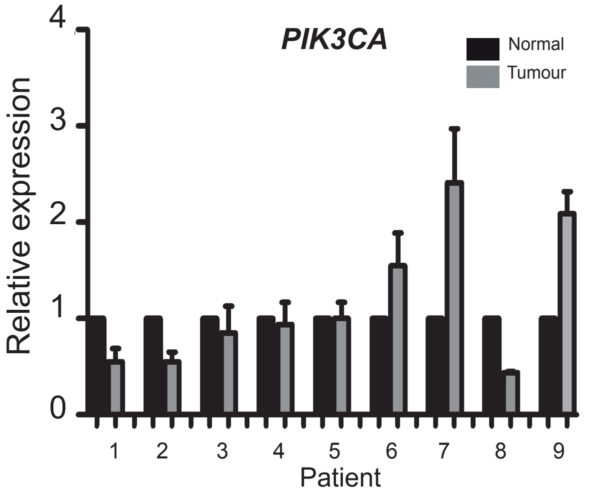

Supplement: Supplementary file 1 [file oncoscience-02-0755-s001.pdf]
